# Identification of Critical miRNAs as Novel Diagnostic Markers for Laryngeal Squamous Cell Carcinoma

**DOI:** 10.1155/2022/6858411

**Published:** 2022-07-20

**Authors:** Kuo Luo, Yanguang Zhao, Hong Liu, Jinhua Mo

**Affiliations:** ^1^Department of Head and Neck Cancer Center, Chongqing University Cancer Hospital, Chongqing 400030, China; ^2^Department of Oncology, Chongqing Hygeia Hospital, Chongqing 400030, China

## Abstract

The prognosis of laryngeal squamous cell carcinoma (LSCC) patients remains poor, and early diagnosis can distinctly improve the long-term survival of LSCC patients. MicroRNAs (miRs) are a group of endogenous, noncoding, 18-24 nucleotide length single-strand RNAs and have been demonstrated to regulate the expression of many genes, thus modulating various cellular biological processes. In this study, we aimed to identify critical diagnostic miRNAs based on two machine learning algorithms. The GSE133632 dataset was acquired from the Gene Expression Omnibus (GEO) datasets, comprising LSCC tissular samples (57 specimens) and matched neighboring healthy mucosa tissular samples (57 specimens). Differentially expressed miRNAs (DEMs) were screened between 57 LSCC specimens and 57 normal specimens. The LASSO regression model and SVM-RFE analysis were carried out for the identification of critical miRNAs. ROC assays were applied to evaluate discriminatory ability. We identified 32 DEMs between LSCC specimens and normal specimens. Two machine learning algorithms confirmed that hsa-miR-615-3p, hsa-miR-4652-5p, hsa-miR-450a-5p, hsa-miR-196a-5p, hsa-miR-21-3p, hsa-miR-139-5p, and hsa-miR-424-5p were critical diagnostic factors. According to the ROC assays, seven miRNAs had an AUC value of >0.85 for LSCC. Taken together, our findings identified seven critical miRNAs in LSCC patients which can be used to diagnose LSCC patients with high sensitivity and specificity. These results must be verified by large-scale prospective studies.

## 1. Introduction

Laryngeal squamous cell carcinoma (LSCC), as one of the most commonly seen tumor types of head and neck malignant cancers, has become a huge burden to the global healthcare system [1]. It is reported that LSCC results in >20,000 mortality in America every year and is considered one of the primary causes of tumor-induced deaths worldwide, particularly in China [2, 3]. Amongst the causes related to the prevalence of LSCC, smoking displays the highest relevance [4]. Moreover, air contamination and chemical contamination can induce LSCC. The 5-year OS of LSCC is >90% with timely intervention; metastases and relapses are the primary factors influencing LSCC prognoses [5, 6]. Poor differentiation and lymph node metastases are associated with reduced five-year survival rate. Hence, it is imperative to develop biomarkers to assist doctors in making correct and timely diagnosis, to forecast clinic results and guide personalized treatment.

MicroRNAs (miRNAs) are noncoding, single-strand RNAs with 20-24 nt long that are extensively distributed in animals and plants [7]. Despite the fact that miRNAs merely take up a small part of the expressed genome, they have been discovered to exert vital effects on a variety of cell processes, like proliferative, apoptotic, and differentiative activities [8, 9]. Previous studies have demonstrated that the aberrant regulation of those tiny and vital molecules in diverse tumor types like LSCC is frequently associated with cancer progression [10]. Apart from their endocellular location and relevant roles, miRNAs can be discovered in the exocellular milieu, specifically fluids such as plasma and serum [11]. The aberrant expressing levels of miRNAs are related to multiple illnesses, and such abnormal regulation makes them underlying markers for illness diagnoses, disease development, and personalized treatment regimens. However, the diagnostic value of miRNAs for LSCC patients remained largely unclear.

In this study, for the first time, we performed machine learning using GSE133632 datasets to identify the sensitive biomarkers for the diagnosis of LSCC. Our findings identified seven critical diagnostic miRNAs which may also be important regulators in the LSCC progression. Our goal is to lay the theoretical basis for further pursuing a novel diagnostic biomarker and a new therapeutic strategy to improve the clinical outcome of LSCC patients.

## 2. Materials and Methods

### 2.1. Microarray Data

The GSE133632 dataset was acquired from the GEO, comprising LSCC tissular samples (57 specimens) and matched neighboring healthy mucosa tissular samples (57 specimens).

#### 2.1.1. Data Processing and Differentially Expressed miRNA (DEM) Screening

The limma package of R was utilized for background correction, normalisation between arrays, and DEM analyses between 57 LSCC and 57 nontumor specimens. Specimens with a modified FDR *P* < 0.05 and |log fold change (FC)| > 1.5 were the liminal values for DEMs.

#### 2.1.2. Establishment of LASSO Model and SVM-RFE Feature Selection Process

The LASSO logistic regressive analyses and the SVM arithmetic were utilized to categorize the diagnosis biomarkers of LSCC [12, 13]. LASSO analyses were completed via the “glmnet” package, the reaction type was binomial, and the alpha was 1. Additionally, as an approach for the purpose of supporting vectors, the Support Vector Machine (SVM) identifies the optimal variates via the deletion of the feature vectors produced by the SVM. The SVM classifier from R package e1071 was utilized for the categorization analyses of the screened biological markers in the diagnoses of AD; *k* = 5 was the setting for the *k*-fold cross-verification, and the parameter of halving was 100.

#### 2.1.3. Diagnosis Significance of Feature Biological Markers in LSCC

For the sake of testing the prediction significance of the determined biological markers, our team produced a ROC curve via the data pertaining to the expressing of mRNAs from 57 LSCC and 57 nontumor specimens. The area under the curve (AUC) value was employed to identify the diagnosis validity in the discrimination of LSCC.

### 2.2. Statistics

Statistic assay was completed via R (v 3.6.3, R Core Team, Massachusetts, USA). Statistical analyses were carried out by the use of either an ANOVA or Student's *t*-test. *P* < 0.05 had significance on statistics.

## 3. Results

### 3.1. Determination of DEMs in LSCC

**Figure 1:**
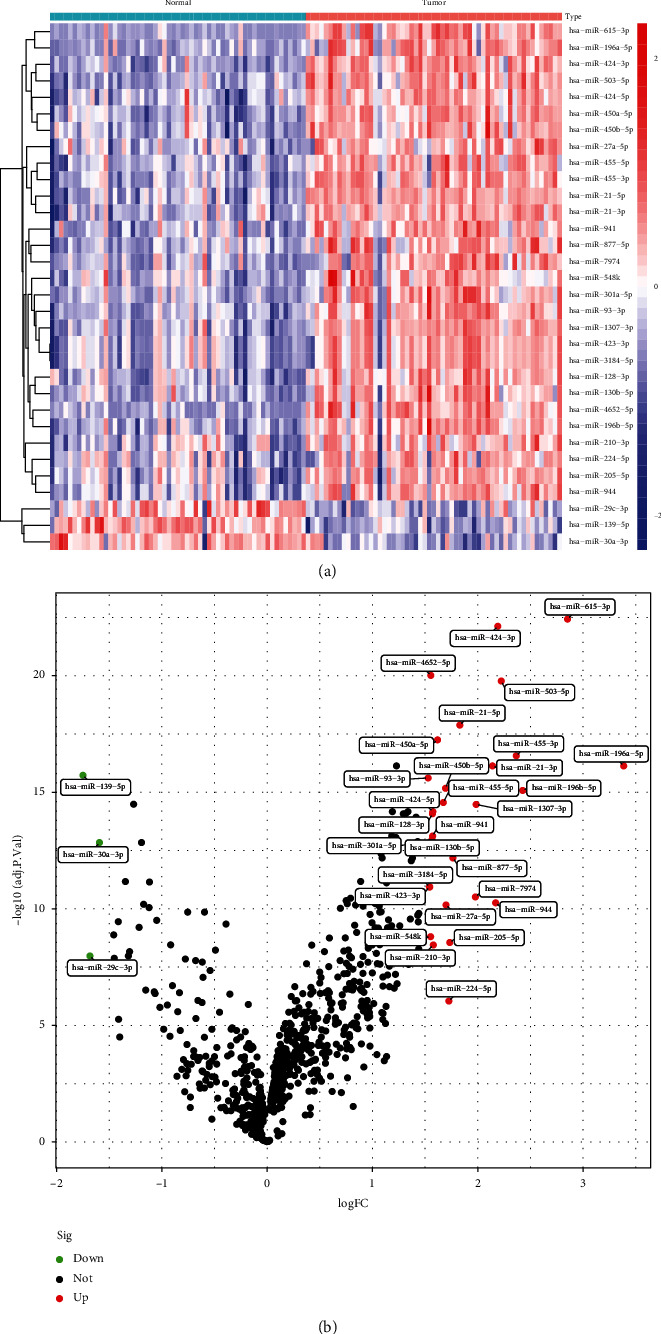
Differentially expressed miRNAs between LSCC samples and nontumor samples in (a) volcano map and (b) heat map.

Data from an overall 57 LSCC and 57 nontumor specimens from GSE133632 datasets were studied herein in a retrospective manner. The DEMs were studied via the limma package. An overall 32 DEMs were acquired: 3 miRNAs were considerably downregulated and 30 miRNAs were considerably upregulated. The DEMs were shown in a heat map (Figure 1(a)) and a volcanic map (Figure 1(b)). Our findings suggested the 32 DEMs may be involved in LSCC progression.

### 3.2. Identification of Diagnostic miRNAs in LSCC

**Figure 2:**
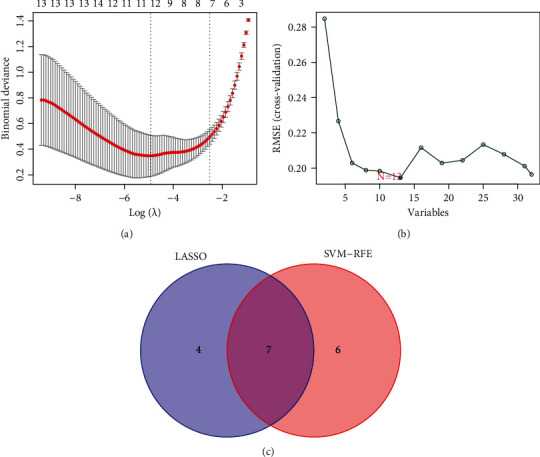
Screening process of diagnostic miRNAs for LSCC diagnosis. (a) Tuning feature selection in LASSO. (b) A plot of biomarker selection via SVM-RFE algorithm. (c) Venn diagram demonstrating seven diagnostic markers shared by the LASSO and SVM-RFE algorithms.

Then, LASSO regressive analyses were utilized to study the DEMs so as to acquire the diagnosis miRNAs from LSCC sufferers. Another approach, like SVM-RFE, was utilized at the same time to select the miRNAs for LSCC diagnoses. Subsequently, our team integrated the LASSO and SVM approaches to acquire the first-rank common miRNAs. The DEMs were identified via the LASSO regressive arithmetic, and we determined 11 miRNAs as diagnosis-related biological markers for LSCC (Figure 2(a)). A subset of 13 miRNAs amongst the DEMs was identified via the SVM-RFE arithmetic (Figure 2(b)). The 7 overlapped miRNAs (hsa-miR-615-3p, hsa-miR-4652-5p, hsa-miR-450a-5p, hsa-miR-196a-5p, hsa-miR-21-3p, hsa-miR-139-5p, and hsa-miR-424-5p) between those 2 arithmetics were eventually chosen (Figure 2(c)). Our findings revealed the 7 overlapped miRNAs should be important regulators.

### 3.3. The Expression and Diagnosis Significance of Seven Overlapping miRNAs in LSCC

**Figure 3:**
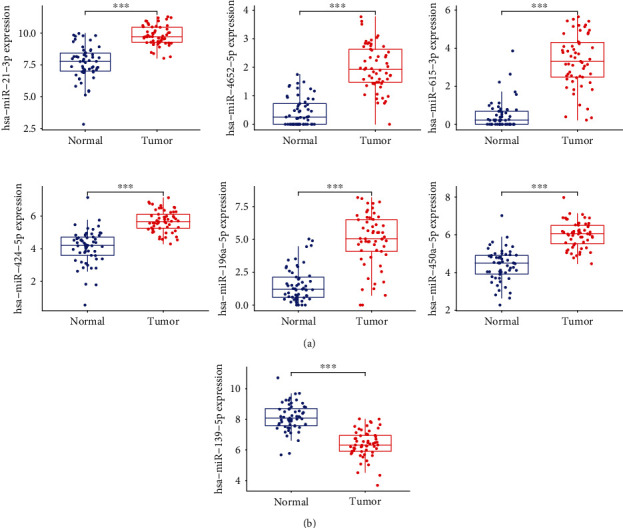
The expressing pattern of seven miRNAs in LSCC. (a) The expression of hsa-miR-615-3p, hsa-miR-4652-5p, hsa-miR-450a-5p, hsa-miR-196a-5p, hsa-miR-21-3p, and hsa-miR-424-5p was distinctly upregulated in LSCC samples vs. healthy samples. (b) The expressing level of hsa-miR-139-5p was considerably downregulated in LSCC samples vs. healthy samples.

**Figure 4:**
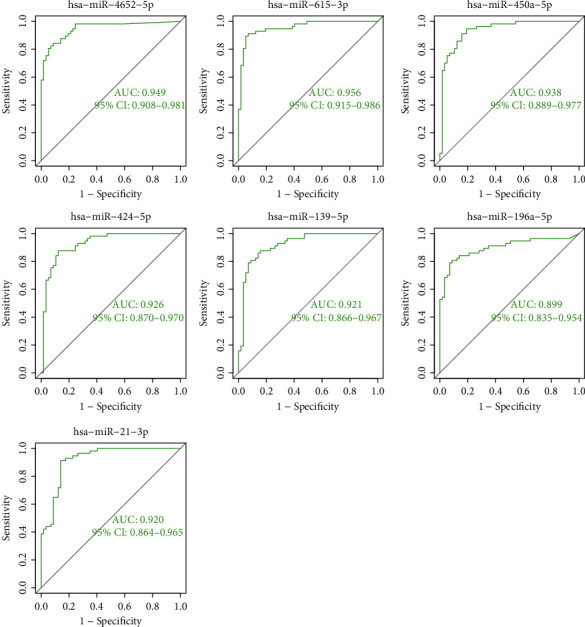
The ROC curve of the diagnostic effectiveness of hsa-miR-615-3p, hsa-miR-4652-5p, hsa-miR-450a-5p, hsa-miR-196a-5p, hsa-miR-21-3p, hsa-miR-139-5p, and hsa-miR-424-5p. The results confirmed their diagnostic value in screening LSCC specimens from nontumor specimens.

Then, our team studied the expressing levels of seven overlapping miRNAs in LSCC and nontumor specimens. As shown in Figure 3(a), the expression of hsa-miR-615-3p, hsa-miR-4652-5p, hsa-miR-450a-5p, hsa-miR-196a-5p, hsa-miR-21-3p, and hsa-miR-424-5p was distinctly upregulated in LSCC samples vs. healthy samples. However, the expressing level of hsa-miR-139-5p was considerably downregulated in LSCC samples vs. healthy samples (Figure 3(b)). In addition, the diagnosis significance of seven critical miRNAs for LSCC sufferers was explored. According to the ROC assays, seven miRNAs had an AUC value of >0.85 for LSCC (Figure 4). Our findings revealed that seven critical miRNAs may be an indicator for the diagnosis of LSCC patients.

## 4. Discussion

LSCC takes up >90% of histologic subtypes of laryngeal cancer [14]. The malignant features, like cancer relapse, metastases, drug tolerance, and chemoradiation tolerance, cause poorer OS and inferior life quality of LSCC sufferers [15]. There is a correlation between aberrant genomic expression and the carcinogenic characteristics of LSCC [16, 17]. In addition, early diagnosis can distinctly improve the clinical outcome of LSCC patients [18, 19]. However, satisfactory diagnostic methods for LSCC have not been achieved. Over the last several years, an increased knowledge pertaining to the molecule-level causal links beneath LSCC initiation and development has provided useful clinical data, not only to its pathogenesis but also to its prognosis and treatment efficacy. Moreover, some novel biomarkers, including miRNAs, have been discovered to be helpful for LSCC diagnosis and prognosis [20, 21].

In this study, we analyzed GSE133632 datasets and identified 32 DEMs. Then, for the first time, we performed LASSO model and SVM-RFE to screen the sensitive diagnostic biomarkers. As new algorithms, they exhibited obvious advantages, and the accuracy of the results is greatly improved. Finally, we identified several diagnostic DEMs, including hsa-miR-615-3p, hsa-miR-4652-5p, hsa-miR-450a-5p, hsa-miR-196a-5p, hsa-miR-21-3p, hsa-miR-139-5p, and hsa-miR-424-5p. Amongst them, the expression of hsa-miR-615-3p, hsa-miR-4652-5p, hsa-miR-450a-5p, hsa-miR-196a-5p, hsa-miR-21-3p, and hsa-miR-424-5p was distinctly upregulated in LSCC specimens vs. healthy specimens. However, hsa-miR-196a-5p expression was distinctly downregulated in LSCC specimens. Previously, their function in LSCC progression is frequent. For instance, Ren et al. reported that miR-21-3p is overexpressed in LSCC and related to late period phases. The suppression of miR-21 via antisense oligonucleotides (ASO) induced the reduced protein content of Ras and the remarkable inhibition of cellular proliferative and invasive abilities [22]. miR-139 expression was distinctly downregulated in LSCC in LSCC patients [23], which was consistent with our findings. In addition, function analysis displayed that ectopic expression of miR-139 repressed cellular proliferative, migratory, and metastatic abilities of LSCC in vitro and in vivo. In addition, the expression and tumor-related function of other DEMs were also reported. In this study, we provided novel clue for the research of the function of miRNAs.

Nevertheless, the present research harbors certain flaws. Firstly, the diagnosis efficiency and prognosis significance of the pivotal miRNAs were merely studied and verified in the GEO datasets. Hence, the outcomes have to be substantiated in more databases. Secondly, the present paper was merely finished as per biological information analyses. For that reason, more assays are necessary to corroborate the outcomes on the foundation of cancer specimens and clinical information. Moreover, in vivo and in vitro assays can reinforce our comprehension of the functions of the pivotal miRNAs in LSCC.

## 5. Conclusion

Overall, we screened seven critical miRNAs based on machine learning. The results in the present paper unveiled that they might harbor comparatively high diagnosis value for LSCC. The discoveries in our research will offer theory-wise enlightenment for investigating the underlying biological markers for the diagnostic and prognostic forecast of LSCC in the future.

## Data Availability

The data used to support the findings of this study are available from the corresponding author upon request.
